# High-Sensitivity Cardiac Troponin T to Exclude Cardiac Involvement in TTR Variant Carriers and ATTRv Amyloidosis Patients

**DOI:** 10.3390/jcm13030810

**Published:** 2024-01-30

**Authors:** Hendrea S. A. Tingen, Milou Berends, Alwin Tubben, Johan Bijzet, Ewout J. Houwerzijl, Friso L. H. Muntinghe, Bart-Jan Kroesen, Paul A. van der Zwaag, Peter van der Meer, Riemer H. J. A. Slart, Bouke P. C. Hazenberg, Hans L. A. Nienhuis

**Affiliations:** 1 Department of Nuclear Medicine and Molecular Imaging, Groningen Amyloidosis Centre of Expertise, University of Groningen, University Medical Centre Groningen, Hanzeplein 1, 9713 GZ Groningen, The Netherlands; 2 Department of Internal Medicine, Groningen Amyloidosis Centre of Expertise, University of Groningen, University Medical Centre Groningen, Hanzeplein 1, 9713 GZ Groningen, The Netherlandsh.l.a.nienhuis@umcg.nl (H.L.A.N.); 3 Department of Cardiology, Groningen Amyloidosis Centre of Expertise, University of Groningen, University Medical Centre Groningen, Hanzeplein 1, 9713 GZ Groningen, The Netherlands; 4 Department of Laboratory Medicine, Groningen Amyloidosis Centre of Expertise, University of Groningen, University Medical Centre Groningen, Hanzeplein 1, 9713 GZ Groningen, The Netherlands; 5 Department of Genetics, Groningen Amyloidosis Centre of Expertise, University of Groningen, University Medical Centre Groningen, Hanzeplein 1, 9713 GZ Groningen, The Netherlands; 6 Biomedical Photonic Imaging Group, Faculty of Science and Technology, University of Twente, Drienerlolaan 5, 7522 NB Enschede, The Netherlands; 7 Department of Rheumatology & Clinical Immunology, Groningen Amyloidosis Centre of Expertise, University of Groningen, University Medical Centre Groningen, Hanzeplein 1, 9713 GZ Groningen, The Netherlands

**Keywords:** amyloidosis, transthyretin, ATTRv, cardiac, cardiomyopathy, carriers, biomarkers, bone scintigraphy, high-sensitivity cardiac troponin T, N-terminal pro B-type natriuretic peptide

## Abstract

(1) Background: Individuals carrying a pathogenic transthyretin gene variant (*TTRv*) are at high risk for developing hereditary transthyretin (ATTRv) amyloidosis and are routinely screened for the development of cardiomyopathy (ATTRv-CM). This study aims to evaluate whether the cardiac biomarkers N-terminal pro B-type natriuretic peptide (NT-proBNP) and high-sensitivity cardiac troponin T (hs-cTnT) can be used to rule out ATTRv-CM. (2) Methods: In this retrospective case-control study, data from 46 ATTRv-CM patients and 101 *TTRv* carriers and ATTRv amyloidosis patients without cardiomyopathy were included. Binary logistic regression models were used to assess the ability of NT-proBNP and hs-cTnT to predict the diagnosis of ATTRv-CM. An optimal cutoff for the relevant biomarker(s) was determined based on a sensitivity of ≥99% and the highest possible percentage of additional tests avoided (%ATA) in the index dataset. (3) Results: Hs-cTnT demonstrated the highest predictive capabilities for ATTRv-CM. The addition of NT-proBNP did not improve the predictive model. A hs-cTnT cutoff of <6 ng/L resulted in a 97% sensitivity and a negative predictive value of 95% with a %ATA of 30% in the validation dataset. (4) Conclusion: In conclusion, hs-cTnT is a useful biomarker for excluding cardiac involvement in *TTRv* carriers and ATTRv amyloidosis patients and it has the potential to prevent unnecessary diagnostic procedures.

## 1. Introduction

Hereditary transthyretin (ATTRv) amyloidosis is caused by pathogenic gene variants in the gene encoding transthyretin (*TTR*). These variants result in the production of unstable transthyretin protein that readily dissociates into misfolded monomers which subsequently aggregate into amyloid fibrils [1]. These amyloid fibrils accumulate in the extracellular spaces of various tissues and organs, primarily leading to neuropathy and/or cardiomyopathy (ATTRv-CM) [2]. The clinical presentation and age at symptom onset can be predicted based on the specific pathogenic variant and family history [2,3]. Currently, there are several treatments available for ATTRv amyloidosis patients, and these are most effective when initiated at an early disease stage [4,5,6]. Several of these treatments are indicated for the treatment of polyneuropathy [7,8,9], and one of these treatments is indicated for cardiomyopathy [10].

Individuals who carry a pathogenic *TTR* gene variant (*TTRv*) are at high risk of developing ATTRv amyloidosis, prompting clinicians to perform regular screening for disease onset in this population. As ATTRv amyloidosis is a systemic disease, screening for the onset of symptoms should target multiple organ systems, necessitating the involvement of an interdisciplinary team of medical specialists. However, as cardiomyopathy is associated with increased mortality in ATTRv amyloidosis patients, this study will focus on screening for the onset of cardiomyopathy [11]. Currently, there is no international consensus on how *TTRv* carriers should be screened [3,12,13,14,15,16,17,18]. Clinical symptoms, cardiac biomarkers, electrocardiograms, and echocardiography are commonly used in screening for cardiac involvement. Additionally, some experts advocate for the use of cardiac magnetic resonance (CMR) imaging [3,19,20]. However, these approaches only detect amyloid deposition once it has already become clinically evident, whereas the objective for *TTRv* carriers should be to detect the onset of subclinical cardiac amyloid deposition to facilitate the early initiation of therapy, and this is an ongoing topic of research [13,18]. Bone scintigraphy and speckle-tracking echocardiography have been used as imaging modalities that could potentially detect ATTRv-CM in its early stages [17,21,22,23,24,25].

Cardiac amyloidosis is typically diagnosed using endomyocardial biopsy or bone scintigraphy [26]. As endomyocardial biopsy has a risk of serious complications and is burdensome for patients, it is not suitable for screening purposes. Grade 1 or higher tracer uptake on bone scintigraphy has demonstrated a sensitivity of 99% and a specificity of 87% in identifying patients with ATTRv-CM [27]. Moreover, as stated above, bone scintigraphy can detect subclinical ATTRv-CM, which could allow for the earlier diagnosis of cardiac involvement, leading to earlier treatment initiation and improved outcomes for ATTRv amyloidosis patients [17,22,23,24,25]. However, it is important to note that lower sensitivity in bone scintigraphy has been reported for some *TTR* gene variants, such as p.Phe84Leu, p.Ser97Tyr, and p.Tyr134Cys, and clinicians should bear this in mind when interpreting bone scintigraphy results [28,29,30,31]. Additionally, bone scintigraphy, in contrast to endomyocardial biopsy, is non-invasive. However, the repetitive use of bone scintigraphy for the screening of *TTRv* carriers implies extra radiation exposure, patient burden, and costs, limiting its frequency of use.

At the Groningen Amyloidosis Centre of Expertise (GrACE) of the University Medical Centre of Groningen (UMCG) in the Netherlands, *TTRv* carriers are invited to the outpatient clinic every two to three years starting from the moment of carrier identification. The frequency of screening visits is increased from ten years prior to the predicted age at disease onset (PADO) based on the pathogenic variant and family history [32]. During cardiac screening visits, we conduct measurements of N-terminal pro B-type natriuretic peptide (NT-proBNP) and high-sensitivity cardiac troponin T (hs-cTnT) and perform electrocardiography and transthoracic echocardiography. Bone scintigraphy and subcutaneous abdominal fat tissue aspirates are routinely performed every two to three years and on indication if there is a suspicion of cardiac involvement. Patients with ATTRv amyloidosis but without cardiac involvement are also systematically followed up in the same way.

Cardiac biomarkers such as NT-proBNP and hs-cTnT are widely used in cardiology to accurately and cost-effectively determine the presence or absence of cardiac pathologies [33,34]. NT-proBNP is released from the heart in response to pressure overload and is used to detect or exclude heart failure [33]. Hs-cTnT is released by cardiomyocytes in response to damage and plays a crucial role in the diagnostic algorithm for identifying myocardial infarction [34]. NT-proBNP and hs-cTnT have proven useful in recognizing cardiac involvement in ATTRv amyloidosis patients, with various cutoffs having been proposed to rule in or rule out ATTRv-CM [11,35,36,37,38]. Nevertheless, the effectiveness of NT-proBNP and hs-cTnT in detecting early-onset subclinical ATTRv-CM in *TTRv* carriers and ATTRv amyloidosis patients remains unclear.

The objective of this study is to investigate the potential of the cardiac biomarkers NT-proBNP and hs-cTnT in predicting diagnosis of ATTRv-CM in *TTRv* carriers and ATTRv amyloidosis patients without cardiomyopathy. We aim to safely exclude cardiomyopathy in *TTRv* carriers and ATTRv amyloidosis patients on the basis of cardiac biomarkers in order to prevent unnecessary diagnostic procedures. 

## 2. Methods

### 2.1. Study Design and Study Population

**Figure 1:**
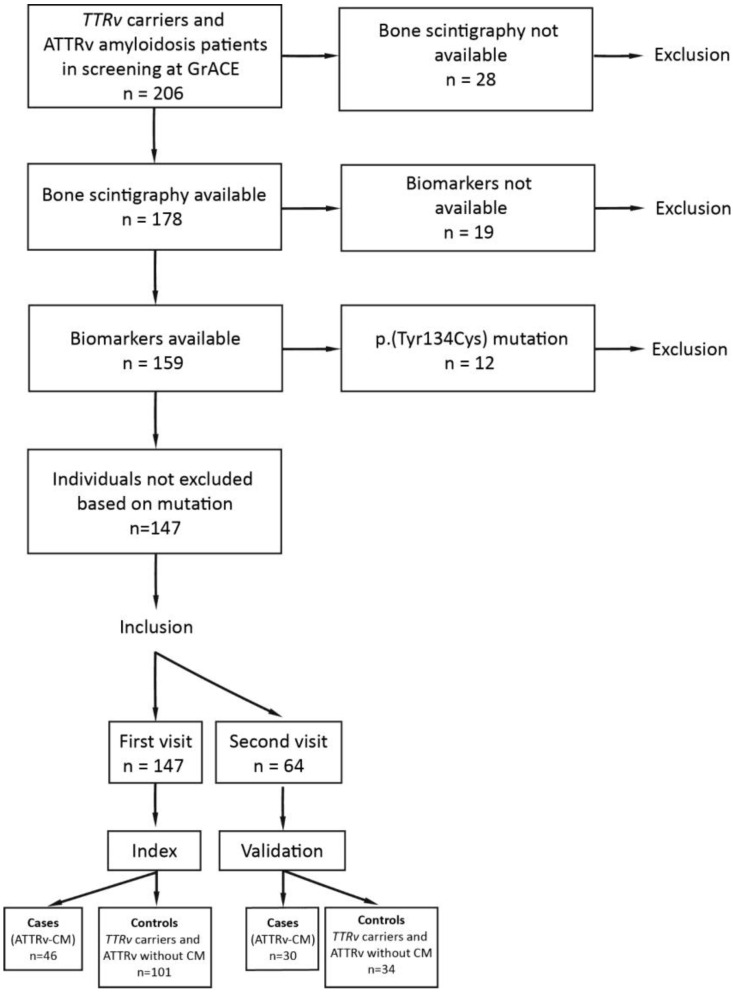
Flowchart of inclusion. *TTRv* = *TTR* gene variant, ATTRv = hereditary transthyretin, GrACE = Groningen Amyloidosis Centre of Expertise, ATTRv-CM = hereditary transthyretin amyloidosis related cardiomyopathy, CM = cardiomyopathy.

In this retrospective case-control study, all individuals with a *TTRv* who were treated or in screening at the GrACE between April 2012 and June 2023 were enrolled. This included ATTRv-CM patients (cases), ATTRv patients without cardiomyopathy (controls), and asymptomatic *TTRv* carriers (controls). As bone scintigraphy was considered the reference standard for ATTRv-CM in this study, individuals carrying a *TTRv* associated with decreased bone scintigraphy sensitivity, such as p.(Tyr134Cys), were excluded [29,30,31]. Visits involving bone scintigraphy and for which NT-proBNP and hs-cTnT were available within one month before or after bone scintigraphy, were used for analysis. An initial analysis was performed on the data of the first visit that met the inclusion criteria for each patient, labelled as the index dataset. Subsequently, an additional dataset, which included the second visit and which met the same inclusion criteria as described before, was generated for each patient, if available. This additional dataset was employed to assess the validity of the specified cutoffs and is referred to as the validation dataset (Figure 1). Because bone scintigraphy is routinely used as a diagnostic procedure in our institution, the potential reduction in its use after applying the newly proposed cutoff was assessed to demonstrate the benefit of this approach.

Diagnoses of ATTRv-CM were based on a positive endomyocardial biopsy, Perugini Grade 2 or 3 cardiac tracer uptake on bone scintigraphy, or Perugini grade 1 cardiac tracer uptake in conjunction with histologically proven amyloid deposits and typical echocardiographic findings as described in the ESC consensus position statement by Garcia-Pavia et al. [20]. CMR imaging was not available for any of the individuals.

All procedures were conducted in compliance with the Declaration of Helsinki. This study was approved by the institutional review board of the UMCG (Registration number: 17471).

### 2.2. Cardiac Biomarkers

The Elecsys NT-proBNP enzyme immunoassay and the Elecsys troponin T high-sensitivity immunoassay (Roche Diagnostics Ltd., Rotkreuz, Switzerland) were utilized to measure plasma NT-proBNP and hs-cTnT, respectively, as part of routine clinical care. All values were extracted from the patient files.

In a previous study at our center, we determined that an NT-proBNP cutoff of >164 ng/L has high sensitivity in detecting ATTRv-CM in *TTRv* carriers [11]. In a multicenter study, a different cutoff of <180 ng/L was proposed to rule out cardiac amyloidosis [35]. For the hs-cTnT assay used at our center, the normal range for healthy adults is below 14 ng/L (99th percentile), and any level above 14 ng/L is considered abnormal for *TTRv* carriers [35,39]. However, based on a prior study, a value below 28.6 ng/L has been identified as optimal for distinguishing ATTRv amyloidosis patients from healthy controls [40]. All cutoffs mentioned above will be assessed in this study.

### 2.3. Bone Scintigraphy

Bone scintigrams were acquired three hours after the intravenous injection of 450–750 MBq [^99m^Tc]Tc-hydroxydiphosphonate (HDP) using dedicated single-photon emission computed tomography/computed tomography (SPECT/CT) systems (Symbia T2, Symbia T16 and Symbia Intevo, Siemens Healthineers, Erlangen, Germany) equipped with a low-energy, high-resolution collimator. Whole-body planar scans were acquired from both posterior and anterior views with a total acquisition time of 12 min (6 min allocated to each view). SPECT images were acquired using a 180° configuration, 64 views, 20 s per view, and a 128 × 128 matrix. The planar images were compared with the SPECT/CT images to rule out blood pool activity. The anterior planar images were assessed based on the Perugini scale [41]. Perugini grade ≥1 was considered abnormal and used as outlined above.

### 2.4. Statistical Analysis

The results are expressed as a median (25th percentile–75th percentile) or as a number (percentage). The baseline characteristics of cases and controls were compared using the Mann–Whitney U test or the Chi Square test where applicable. A logistic regression analysis was used to evaluate the value of NT-proBNP and hs-cTnT in predicting diagnosis of ATTRv-CM and bone scintigraphy outcome (abnormal vs. normal).

The diagnostic performance of the most promising biomarker or combination of biomarkers, determined through logistic regression, was further assessed in predicting diagnosis of ATTRv-CM and bone scintigraphy outcome using a receiver operating characteristic (ROC) analysis. The area under the ROC curve (AUC) was calculated to quantify the discriminative ability of the biomarker(s) in predicting diagnosis of ATTRv-CM or the bone scintigraphy findings.

Additionally, an extensive analysis was conducted to explore all possible combinations of NT-proBNP and hs-cTnT to assess their diagnostic accuracy in predicting diagnosis of ATTRv-CM. Each biomarker combination with values equal to or exceeding the proposed cutoff values for NT-proBNP and/or hs-cTnT was considered indicative of a positive outcome. The true positive rate, true negative rate, positive predictive value, and negative predictive value (NPV) were computed for each potential biomarker combination. Additionally, the percentage of individuals in whom the biomarker(s) fell below the specified NT-proBNP and/or hs-cTnT cutoffs, which corresponded with the percentage of individuals in whom additional testing could have been avoided (%ATA), was calculated. The selection of the optimal cutoff value for the biomarker(s) was based on achieving a sensitivity equal to or exceeding 99%, along with maximizing the %ATA. Additionally, to improve the interpretability of the newly proposed cutoff, the diagnostic accuracy of the currently employed cutoffs was assessed within our cohort. Finally, we assessed the validity of the newly proposed cutoff by applying it to the validation dataset.

Analyses were performed using SPSS version 28 (IBM Corp, Armonk, NY, USA) and R-studio version 4.3.1 (R Foundation for Statistical Computing, Vienna, Austria). The level of significance for all hypothesis tests was set at 0.05.

## 3. Results

### 3.1. Clinical and Demographic Characteristics

A total of 206 individuals with a *TTRv* were evaluated at GrACE between April 2012 and June 2023. In total, 28 subjects were excluded based on the unavailability of bone scintigraphy images. From the remaining 178 individuals, biomarkers were not available in 19 individuals. Twelve individuals were excluded based on having the p.(Tyr134Cys) variant, which indicates a risk of a false negative bone scintigram. The remaining 147 individuals were included for further analysis in the index dataset. Of the individuals in the index dataset, 46 individuals (31%) had ATTRv-CM, and 101 individuals (69%) were either *TTRv* carriers or ATTRv amyloidosis patients without cardiomyopathy. The validation dataset consisted of 64 individuals, of whom 30 individuals had ATTRv-CM (47%). A flow chart of inclusion is shown in Figure 1.

In the index dataset, differences in age, sex, wall thicknesses on echocardiograms, NT-proBNP, hs-cTnT, and Perugini grade were observed between cases and controls. The baseline characteristics are provided in Table 1.

### 3.2. Prediction of Diagnosis of ATTRv-CM

#### 3.2.1. Logistic Regression Outcomes

In the univariable logistic regression, both hs-cTnT and NT-proBNP emerged as significant predictors of diagnosis of ATTRv-CM (Table 2). In the multivariable logistic regression, only hs-cTnT retained its significance, while NT-proBNP no longer contributed to the model (Table 2).

#### 3.2.2. ROC Analysis for hs-cTnT

**Figure 2:**
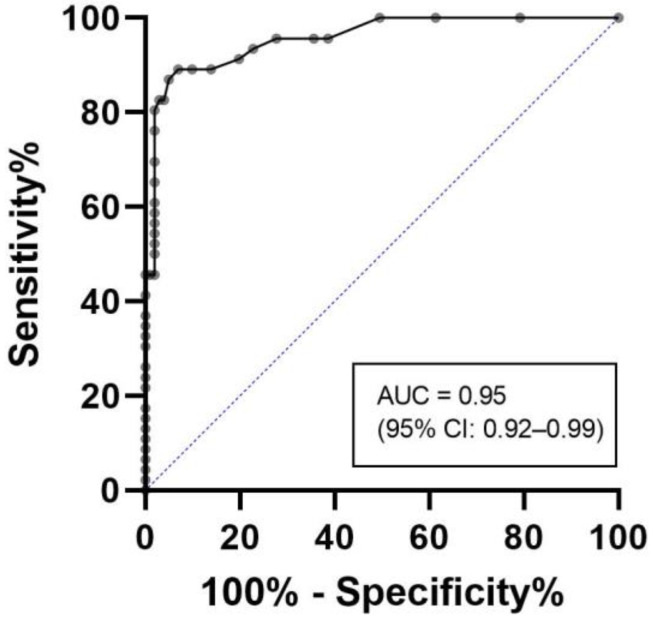
ROC analysis of the predictive value of hs-cTnT for ATTRv-CM. ROC = receiver operator characteristics, hs-cTnT = high-sensitivity cardiac troponin T, ATTRv-CM = hereditary transthyretin amyloidosis related cardiomyopathy, AUC = area under the curve.

As hs-cTnT was the only significant biomarker in the multivariable logistic regression, an ROC analysis was subsequently performed exclusively for hs-cTnT to assess its diagnostic performance, and the results are shown in Figure 2. The AUC for hs-cTnT in predicting the diagnosis was 0.95 (95% confidence interval (CI): 0.92–0.99), indicating good discriminative ability.

#### 3.2.3. hs-cTnT Cutoff to Predict Diagnosis of ATTRv-CM

**Figure 3:**
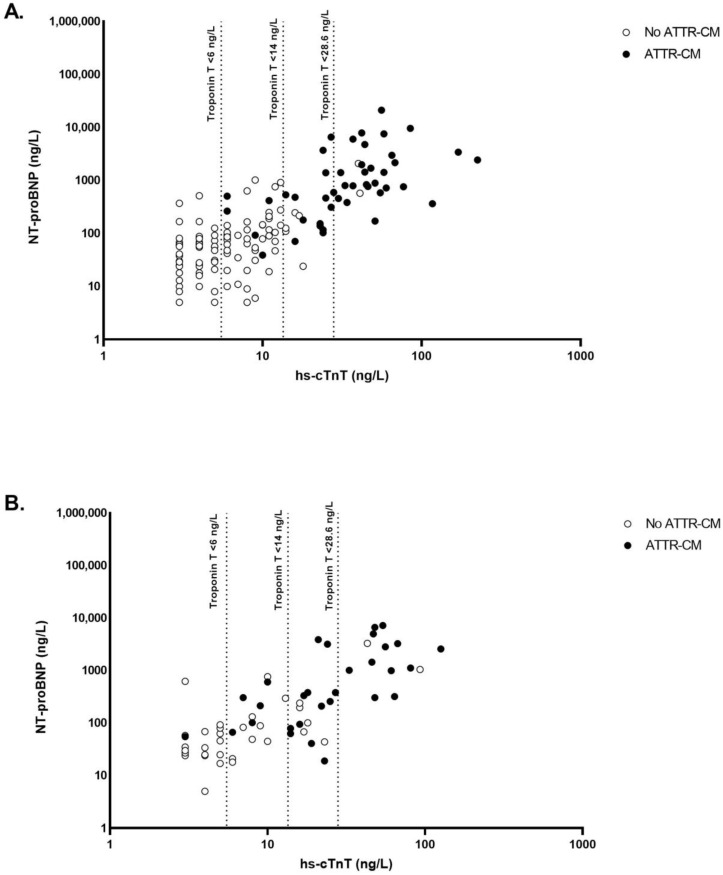
Diagnostic accuracy of various cutoffs of hs-cTnT to predict ATTRv-CM in the index dataset (**A**) and the validation dataset (**B**). hs-cTnT = high-sensitivity cardiac troponin T, ATTRv-CM = hereditary transthyretin amyloidosis related cardiomyopathy.

The optimal cutoff for hs-cTnT to predict the absence of ATTRv-CM was <6 ng/L, which yielded a sensitivity of 100% with no false negatives and an NPV of 100% (Figure 3A and Table 3). In 51 patients (35%), the hs-cTnT was below the proposed cutoff. Utilizing hs-cTnT as a criterion in screening could have prevented these patients from undergoing additional investigations without missing any of the ATTRv-CM patients. In Figure 3A, the currently used cutoffs of <14 ng/L and <28.6 ng/L are shown together with the newly proposed cutoff of <6 ng/L. Employing the cutoff of <14 ng/L resulted in 5 missed ATTRv-CM patients (11%), and had the cutoff of 28.6 ng/L been employed, 19 ATTRv-CM patients (41%) would have been missed (Table 3).

#### 3.2.4. Performance of hs-cTnT Cutoff <6 ng/L in Validation Dataset 

The diagnostic accuracy of the newly suggested cutoff was evaluated in the validation dataset. In this dataset, the hs-cTnT cutoff of <6 ng/L resulted in a sensitivity of 97%. One ATTRv-CM patient had a false negative result, and the NPV was 95%. Additional testing or imaging would have been avoided in 19 individuals (30%) (Figure 3B and Table 3). Using the hs-cTnT cutoffs of <14 ng/L and <28.6 ng/L resulted in 6 and 18 ATTRv-CM patients being missed, respectively (Table 3).

### 3.3. Prediction of Bone Scintigraphy Outcome

The above analyses were repeated for the prediction of ATTRv-CM based on a positive bone scintigram (Perugini grade 1 or higher). This analysis was performed to gain further insights into what might be overlooked if no additional imaging is performed in individuals with low cardiac biomarkers. As with the results observed in the model predicting the diagnosis of ATTRv-CM, both NT-proBNP and hs-cTnT were significant predictors of bone scintigraphy outcome in the univariable logistic regression analysis, and only hs-cTnT remained a significant predictor in the multivariable logistic regression analysis (Table S1). The ROC analysis revealed good discriminative ability with an AUC of 0.95 (95% CI: 0.91–0.98) (Figure S1). To predict bone scintigraphy outcome, the optimal hs-cTnT cutoff was determined to be <6 ng/L, which resulted in a sensitivity of 100% with no false negatives and a negative predictive value (NPV) of 100% (Figure S2). Implementing this cutoff would have prevented bone scintigrams in 51 patients (35%). The diagnostic accuracy of this cutoff, along with the two currently employed cutoffs, is shown in Table S2. Applying the hs-cTnT cutoff of <6 ng/L to the validation dataset resulted in a sensitivity of 94%, with two false negative cases and an NPV of 89%. Employing this cutoff would have prevented additional testing or imaging in 18 individuals (30%) (Figure S2). 

## 4. Discussion

This study shows that hs-cTnT can be used to rule out ATTRv-CM with high sensitivity and a high NPV in *TTRv* carriers and ATTRv amyloidosis patients. The addition of NT-proBNP or a combination of NT-proBNP and hs-cTnT does not improve the selection process. The use of this hs-cTnT cutoff in a two-step screening approach, wherein additional tests are ordered based on the hs-cTnT level, could have resulted in a 30% reduction in the number of bone scintigrams performed at our center.

The aim of this study was to evaluate whether ATTRv-CM can be safely excluded in *TTRv* carriers and ATTRv amyloidosis patients using two established cardiac biomarkers. To minimize the number of missed ATTRv-CM patients, we selected a cutoff to achieve high sensitivity and a high NPV, with specificity considered of secondary importance. The optimal hs-cTnT cutoff was determined to be 6 ng/L. With this cutoff, no ATTRv-CM patients were missed in the index dataset. Within the validation dataset, one ATTRv-CM patient had an hs-cTnT level below the cutoff and would have been missed if hs-cTnT was used in screening. This case concerns a newly identified ATTRv-CM patient with an hs-cTnT measurement of 3 ng/L, no cardiac symptoms, and no abnormalities on their echocardiogram. One and a half years later, their hs-cTnT exceeded the 6 ng/L cutoff, so this would have led to a diagnostic delay of one and a half years if hs-cTnT had been used in screening. Although this asymptomatic case initially would have been missed, the same cutoff level did enable detection at a later moment, and so reduced eventual harm to the patient. Therefore, a sensitivity of 97% instead of >99% was felt to be acceptable.

Our hs-cTnT cutoff level differs from previously established cutoff levels for hs-cTnT. However these cutoff levels were established in different patient populations, namely in patients with suspected cardiac amyloidosis, and with the aim to obtain a high sensitivity as well as the highest possible specificity [35,40]. Our data show that the previously proposed hs-cTnT cutoffs of 14 ng/L and 28.6 ng/L are inadequate for excluding ATTRv-CM in *TTRv* carriers with or without amyloidosis. For instance, using the cutoff of 14 ng/L yields a sensitivity of 89% and an NPV of 95% in our index dataset, and this would have resulted in five patients with ATTRv-CM being missed.

In addition to assessing the predictive value of hs-cTnT for ATTRv-CM, we also examined its predictive value for the outcome of bone scintigraphy, given that any grade of cardiac tracer uptake on bone scintigraphy could be considered relevant in *TTRv* carriers as it concerns a population with a high a priori probability of developing ATTRv-CM and could potentially indicate the subclinical onset of ATTRv-CM. As with the prediction of ATTRv-CM, the optimal hs-cTnT cutoff for predicting bone scintigraphy outcome in the index dataset was determined to be 6 ng/L, with no false negatives. Implementing this cutoff to preselect individuals for further diagnostic testing would have prevented bone scintigraphy in 51 patients (35%) at our center. In the validation dataset, two false negatives (Perugini grade 1 and 3) occurred, while bone scintigraphy would have been avoided in 30% of the patients.

NT-proBNP and hs-cTnT levels increase with advancing age, and they are higher in males and patients with renal insufficiency [42,43,44,45]. These potential confounders are particularly relevant in relation to the specificity of NT-proBNP and hs-cTnT cutoffs as they could lead to some *TTRv* carriers or ATTRv amyloidosis patients being wrongly selected for additional testing for cardiac involvement. However, it is important to note that our study was not focused on accurately identifying ATTRv-CM patients, but rather on effectively excluding ATTRv-CM. Therefore, the influence of these confounding factors on our results is limited, and the likelihood of missing ATTRv-CM patients due to confounder effects was deemed low.

Selecting *TTRv* carriers for additional testing based on hs-cTnT could bring about improvements for both patients and the healthcare system, particularly at centers where bone scintigraphy is performed routinely. Firstly, by preselecting *TTRv* carriers using the hs-cTnT cutoff, a reduction in radiation exposure could be achieved as each bone scintigram involves a radiation dose of approximately 3.35 mSv when a SPECT/CT is performed and 500 MBq of [^99m^Tc]Tc-HDP is administered [46]. Although this radiation dose is small, the cumulative effect of repetitive exposure in the screening program has not been established [25]. Additionally, reducing the number of bone scintigrams performed would decrease the burden on *TTRv* carriers and the burden on the healthcare system. Lastly, the implementation of the hs-cTnT cutoff would reduce the cost of the healthcare system. Importantly, using the hs-cTnT cutoff reliably rules out ATTR-CM, eliminating the need for any further screening tests, including echocardiography and CMR imaging. Therefore, selecting *TTRv* carriers for additional testing based on hs-cTnT would not only reduce the number of unnecessary bone scintigrams, but also unnecessary echocardiograms and CMRs.

Our study has some limitations. Firstly, the relatively small population size and the absence of validation in an external cohort diminish the generalizability of our findings. Hence, we recommend assessing the suggested cutoff of 6 ng/L in an external cohort, especially considering documented cases in the literature where hs-cTnT falls below 6 ng/L in ATTRv-CM patients [25]. Additionally, it is important to note that NPV inherently varies with pre-test probability, and that therefore the NPV of the determined cutoff only applies to a cohort of *TTRv* carriers and ATTRv amyloidosis patients. Furthermore, due to the exclusion of individuals carrying the p.(Tyr134Cys) variant, the performance of the determined cutoff in these individuals is uncertain. Moreover, the retrospective case-control design of the study could have introduced bias and led to an overestimation of the diagnostic performance of the cutoff and could additionally have led to an absence of CMR imaging in all individuals and an absence of echocardiograms in two thirds of individuals. Another drawback inherent to the case-control design is that treatment had been initiated in ATTRv-CM patients. Heart failure therapy may have influenced cardiac biomarker levels, particularly NT-proBNP levels [47,48,49,50,51]. Furthermore, rate control therapy for patients with atrial fibrillation might have contributed to a reduction in hs-cTnT levels [52].

## 5. Conclusions

A cutoff of 6 ng/L for hs-cTnT can identify *TTRv* carriers and ATTRv amyloidosis patients in whom ATTRv-CM is very unlikely with high sensitivity and a high NPV. Using this threshold to preselect *TTRv* carriers for additional diagnostic testing could spare 30% of patients from unnecessary testing. This reduction in the number of diagnostic tests has the potential to enhance screening for cardiac involvement in *TTRv* carriers, offering benefits to both patients and the healthcare system.

## Figures and Tables

**Table 1:** Patient characteristics at baseline.

	Cases (ATTRv-CM)	Controls (No ATTRv-CM)	*p*-Value
**Number of patients**	46	101	
**Age (years)**	67 (58–71)	52 (42–61)	<0.001 *
**Male**	36 (78%)	45 (45%)	<0.001 *
**Treatment**			
**Disease-specific treatment**			
▪ Patisiran	1 (2%)	1 (1%)	ns
▪ TTR stabilizer			ns
- Diflunisal 250 mg twice daily	5 (11%)	10 (10%)	
- Tafamidis			
▪ 20 mg daily	3 (7%)	6 (6%)	
▪ 80 mg daily	2 (4%)	0 (0%)	
**Heart failure treatment**			
▪ Diuretics	16 (35%)	12 (12%)	0.001 *
▪ Dihydropyridine	4 (9%)	6 (6%)	ns
▪ Calcium antagonist	0 (0%)	1 (1%)	ns
▪ Angiotensin receptor blocker	5 (11%)	7 (7%)	ns
▪ Angiotensin converting enzyme inhibitor	9 (20%)	13 (13%)	ns
▪ Beta blocker	14 (30%)	8 (8%)	<0.001 *
**Number of transthoracic echocardiograms**	22 (48%)	27 (27%)	
**Wall thickness on echocardiogram**			
**Interventricular septal wall thickness (mm)**	15 (10–17)	10 (9–12)	0.002 *
**Left ventricular posterior wall thickness (mm)**	13 (10–15)	9 (8–10)	<0.001 *
**NT-proBNP (ng/L)**	767 (352–2241)	62 (28–114)	<0.001 *
**hs-cTnT (ng/L)**	36 (24–55)	5 (4–9)	<0.001 *
**eGFR (mL/min/1.73 m^2^)**	89 (73–95)	89 (72–103)	ns
**Mutation**			ns
**p.(Val50Met)**	20 (44%)	68 (67%)	
**p.(Val142Ile)**	8 (17%)	10 (10%)	
**p.(Val91Ala)**	6 (13%)	3 (3%)	
**p.(Glu109Lys)**	3 (7%)	4 (4%)	
**p.(Val114Ala)**	1 (2%)	6 (6%)	
**Other**	7 (17%)	10 (10%)	
**Perugini Grade**			<0.001 *
**0**	0 (0%)	92 (91%)	
**1**	1 (2%)	9 (9%)	
**2**	17 (37%)	0 (0%)	
**3**	28 (61%)	0 (0%)	

Values are median (25th and 75th percentile) or number of patients (percentage). NT-proBNP = N-terminal pro B-type natriuretic peptide, hs-cTnT = high-sensitivity cardiac troponin T, eGFR = estimated glomerular filtration rate, ATTRv-CM = hereditary ATTR amyloidosis with cardiomyopathy, ns = not significant, * = significant.

**Table 2:** Univariable and multivariable logistic regression analysis for predicting ATTRv-CM in the index dataset.

	Univariable Analysis			Multivariable Analysis		
Variable	OR	95% CI	*p*-Value	OR	95% CI	*p*-Value
**NT-proBNP (ng/L)**	1.003	1.002–1.005	<0.001 *	1.000	0.999–1.001	0.457
**hs-cTnT (ng/L)**	1.196	1.123–1.274	<0.001 *	1.177	1.097–1.263	<0.001 *

TTRv = transthyretin gene variant, OR = odds ratio, 95% CI = 95% confidence interval, NT-proBNP = N-terminal pro B-type natriuretic peptide, hs-cTnT = high-sensitivity cardiac troponin T, * = significant variable.

**Table 3:** Diagnostic performance of various hs-cTnT cutoffs in predicting ATTRv-CM.

	Sens	NPV	FN	Spec	PPV	FP	n ATA	%ATA	Sens	NPV	FN	Spec	PPV	FP	n ATA	%ATA
**<6 ng/L**	100%	100%	0	50%	48%	50	51	35%	97%	95%	1	53%	64%	16	19	30%
**<14 ng/L**	89%	95%	5	93%	85%	7	99	68%	80%	82%	6	79%	77%	7	33	52%
**<28.6 ng/L**	59%	84%	19	98%	93%	2	118	80%	40%	64%	18	94%	86%	2	50	78%

hs-cTnT = high-sensitivity cardiac troponin T, Sens = sensitivity, NPV = negative predictive value, FN = false negatives, Spec = specificity, PPV = positive predictive value, FP = false positives, n ATA = number of patients in whom additional tests are avoided, %ATA = percentage of patients in whom additional tests are avoided.

## Data Availability

The datasets generated and/or analyzed in the current study are available from the corresponding author on reasonable request.

## Supplementary Materials

The following supporting information can be downloaded at: https://www.mdpi.com/article/10.3390/jcm13030810/s1, Table S1: Univariable and multivariable logistic regression analysis for predicting bone scintigraphy outcome in the index dataset. Table S2: Diagnostic performance of various hs-cTnT cutoffs to predict bone scintigraphy outcome. Figure S1: ROC analysis of the predictive value of hs-cTnT for bone scintigraphy outcome. Figure S2: Diagnostic accuracy of various cutoffs of hs-cTnT to predict bone scintigraphy outcome in the index dataset (A) and the validation dataset (B).

## Abbreviations

%ATA	Percentage of additional tests avoided
ATTRv	Hereditary transthyretin
ATTRv-CM	ATTRv-related cardiomyopathy
AUC	Area under the curve
CI	Confidence interval
CMR	Cardiac magnetic resonance
GrACE	Groningen Amyloidosis Centre of Expertise
HDP	Hydroxydiphosphonate
hs-cTnT	High-sensitivity cardiac troponin T
NPV	Negative predictive value
NT-proBNP	N-terminal pro B-type natriuretic peptide
ROC	Receiver operating characteristics
SPECT/CT	Single-photon emission computed tomography/computed tomography
PADO	Predicted age at disease onset
*TTR*	Transthyretin gene
*TTRv*	Pathogenic transthyretin gene variant
UMCG	University Medical Centre Groningen
