# Supplementary material for: High-Sensitivity Cardiac Troponin T to Exclude Cardiac Involvement in TTR Variant Carriers and ATTRv Amyloidosis Patients
Source: J Clin Med. 2024 Jan 30;13(3):810. doi: 10.3390/jcm13030810 (PMC10856062; doi:10.3390/jcm13030810)
Supplement: Supplementary file 1 [file jcm-13-00810-s001.zip › Supplemental tables and figures.pdf]

High-Sensitivity Cardiac Troponin T to Exclude Cardiac Involvement in TTR Variant Carriers and ATTRv Amyloidosis Patients

Hendrea S. A. Tingen, Milou Berends, Alwin Tubben, Johan Bijzet, Ewout J. Houwerzijl, Friso L. H. Muntinghe, Bart-Jan Kroesen, Paul A. van der Zwaag, Peter van der Meer, Riemer H. J. A. Slart, Bouke P. C. Hazenberg and Hans L. A. Nienhuis

Supplemental tables and figures

Table S1. Univariable and multivariable logistic regression analysis for predicting bone scintigraphy outcome in the index dataset.

	Univariable analysis			Multivariable analysis		
**Variable**	**OR**	**95% CI**	**p-value**	**OR**	**95% CI**	**p-value**
**NT-proBNP (ng/L)**	1.005	1.003-1.007	<.001*	1.001	0.998-1.003	.600
**hs-cTnT (ng/L)**	1.353	1.201-1.523	<.001*	1.335	1.178-1.514	<.001*

*TTRv = transthyretin gene variant, OR = odds ratio, 95% CI = 95% confidence interval, NT-proBNP = N-terminal pro B-type natriuretic peptide, hs-cTnT =high-sensitivity cardiac troponin T, * = significant variable*

Table S2. Diagnostic performance of various hs-cTnT cutoffs to predict bone scintigraphy outcome.

Hs-cTnT cutoff	**Index dataset**								**Validation dataset**							
	Sens	*NPV*	*FN*	*Spec*	*PPV*	*FP*	*n ATA*	*%ATA*	*Sens*	*NPV*	*FN*	*Spec*	*PPV*	*FP*	*n ATA*	*%ATA*
**<6 ng/L**	100%	100%	0	55%	57%	41	51	35%	94%	89%	2	59%	73%	12	19	30%
**<14 ng/L**	80%	89%	11	96%	92%	4	99	67%	74%	73%	9	73%	84%	5	33	52%
**<28.6 ng/L**	53%	78%	26	100%	100%	0	118	80%	40%	58%	21	58%	100%	0	50	78%

Hs-cTnT = high-sensitivity cardiac troponin T, Sens = sensitivity, NPV = negative predictive value, FN = false-negatives, Spec = specificity, PPV = positive predictive value, FP = false-positives, n ATA = number of patients in whom additional tests are avoided, %ATA = percentage of patients in whom additional tests are avoided.

Supplemental figures and captions

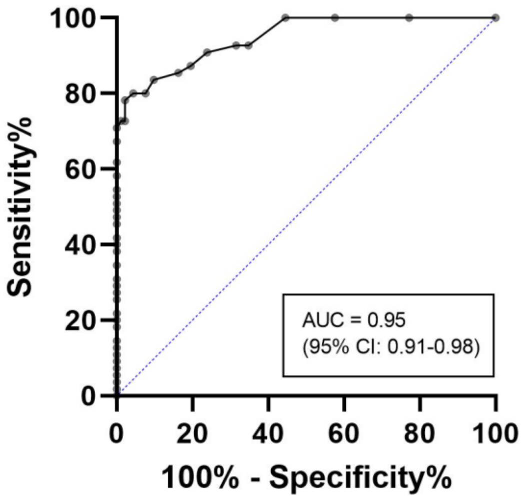

Figure S1. ROC analysis of the predictive value of hs-cTnT for bone scintigraphy outcome.

ROC = receiver operator characteristics, hs-cTnT = high-sensitivity cardiac troponin T, AUC = area under the curve.

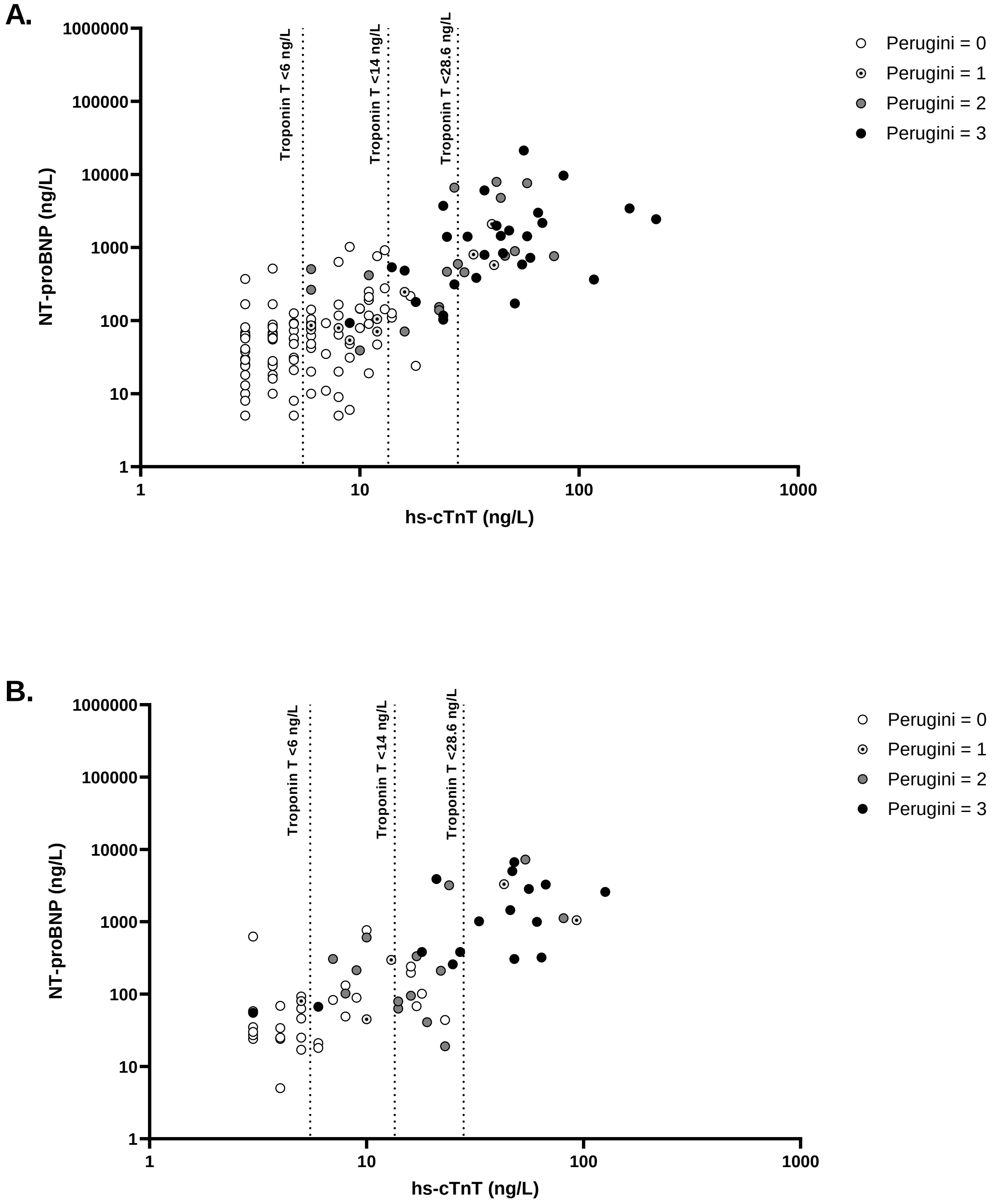

Figure S2. Diagnostic accuracy of various cutoffs of hs-cTnT to predict bone scintigraphy outcome in the index dataset (A) and the validation dataset (B).

hs-cTnT = high-sensitivity cardiac troponin T.
